# Trophoblastic Infiltration in Tubal Pregnancy Evaluated by Immunohistochemistry and Correlation with Variation of Beta-Human Chorionic Gonadotropin

**DOI:** 10.1155/2014/302634

**Published:** 2014-01-09

**Authors:** Danyelle Farias Ferreira, Julio Elito Júnior, Edward Araujo Júnior, João Norberto Stavale, Luiz Camano, Antonio Fernandes Moron

**Affiliations:** ^1^Department of Obstetrics, São Paulo Federal University (UNIFESP), Rua Carlos Weber 956, Apartamento 113 Visage, Alto da Lapa, São Paulo, SP, Brazil; ^2^Department of Pathology, São Paulo Federal University (UNIFESP), São Paulo, SP, Brazil

## Abstract

*Objective*. To evaluate trophoblastic cell proliferation and angiogenesis in tubal pregnancy assessed by immunohistochemical study and their correlation with an average variation of **β**-hCG in an interval of 48 hours before surgery. *Methods*. A prospective study was conducted on 18 patients with a diagnosis of tubal pregnancy. The patients were divided into two groups of ectopic pregnancy of which 11 showed rise of **β**-hCG levels and 7 patients showed declining **β**-hCG levels in an interval of 48 hours prior to surgery. Trophoblastic cell proliferation and angiogenesis were assessed by Ki-67 and VEGF, respectively. Trophoblastic cell proliferation was assessed by Ki-67 and was classified into three groups (grade I: less than 1/3 of stained nuclei, grade II: 1/3 to 2/3 of the stained nuclei, and grade III: more than 2/3 of the nuclei stained). The cases analyzed for VEGF were divided into three groups (grade I: less than 1/3 of the stained cytoplasm; grade II: 1/3 to 2/3 of the stained cytoplasm; grade III: more than 2/3 of the stained cytoplasm). Statistical analysis was performed using the chi-square, ANOVA, and Kruskal-Wallis tests. *Results*. The mean variation in the serum **β**-hCG levels in 48 hours in tubal pregnancy patients correlated with trophoblastic cell proliferation assessed by Ki-67 and showed a decline of 13.46% in grade I, a rise of 45.99% in grade II, and ascension of 36.68% in grade III (*P* = 0.030). The average variation in the serum **β**-hCG in 48 hours, where angiogenesis was evaluated by VEGF, showed a decline of 18.35% in grade I, a rise of 32.95% in grade II, and ascension of 37.55% in grade III (*P* = 0.047). *Conclusions*. Our observations showed a direct correlation of increased levels of serum **β**-hCG in 48h period prior to surgery with higher trophoblastic cell proliferation assessed by Ki-67 and angiogenesis assessed by VEGF in tubal pregnancy.

## 1. Introduction

An ectopic pregnancy is any pregnancy where the embryo is implanted outside the uterus cavity and tubal pregnancy is when the embryo is implanted in a fallopian tube. Hence, tubal pregnancy is one type of ectopic pregnancy. Ectopic pregnancy (EP) is the leading cause of maternal mortality in the first trimester of pregnancy resulting from acute abdominal bleeding [1, 2]. It is important to find mechanisms that are able to predict indirectly the risk of tubal rupture. EP presents a broad clinical spectrum; some cases progress to healing spontaneously, while other cases result in tubal rupture.

Treatment can be surgical (radical or conservative), clinical with methotrexate (MTX), or expectant management. In cases of tubal rupture, the classical treatment is salpingectomy, while conservative surgery is indicated in unruptured tubal pregnancy and is desirable to maintain fertility.

The likely cause for the difference in outcome of cases is related to the degree of infiltration of trophoblast in the fallopian tube; thus the study of infiltration modulators allows an understanding of the pathological processes of EP [3]. The immunohistochemical study enables understanding and quantifying the distribution and location of markers and proteins that are expressed in trophoblast, allowing the recognition of the relationship between maternal and trophoblastic tissues.

Studies have shown that at least two processes are closely related to trophoblastic infiltration: the first is related to the proliferative capacity of the cells and the second is related to the angiogenic action at the site of implantation [4–8].

The proliferative activity of any tissue can be determined by the cell proliferation process resulting from the cascade of events which ensures cell division and replication [9–11]. The Ki-67 antibody recognizes proteins of the cell nucleus and is a marker for differentiating different phases of mitotic division, ensuring accurate measurement of the number of proliferating cells [11].

Vascular endothelial growth factor (VEGF) is produced and secreted under hypoxic [6] conditions and can also be induced by other growth factors and cytokines. The tubal environment is very different from endometrium as it is much more vascularized, and this may affect the synthesis and release of VEGF. Possibly, the implementation of the conceptus in the tube can stimulate the production of VEGF in an attempt to accommodate the pregnancy in this unfavorable environment. A study has demonstrated that higher levels of VEGF in the early stages of pregnancy could prove to be a potential serum marker for EP [6].

The objective of this study was to correlate the serum *β*-hCG levels in 48-hour period prior to surgery with immunohistochemical analysis using Ki-67 to assess trophoblastic cell proliferation and VEGF to assess angiogenesis in tubal pregnancy.

## 2. Material and Methods

We conducted a prospective, descriptive, and observational study of 18 patients with EP from January 2011 to March 2012. This study was approved by the Ethics Committee of the Federal University of São Paulo (UNIFESP) under number 1986/08; signed consent forms were obtained from patients participating in this study.

Inclusion criterion was diagnosis of unruptured tubal pregnancy with rising or declining levels of *β*-hCG between two measurements in an interval of the onset of tubal pregnancy at the end of 48 hours before surgery. Exclusion criteria were nonrepresentative sample for immunohistochemical analysis and nontubal ectopic pregnancy. The patients enrolled in this study were from a tertiary care hospital of UNIFESP. All patients with unruptured tubal pregnancy on whom two measurements of serum *β*-hCG levels were determined in an interval of 48 hours were invited to participate in this study. Some of the invited patients chose not to participate and were referred to nonsurgical treatment (expectant management or medical treatment with methotrexate (MTX)). Thus, the selection of the study participants was random. In this study participants were divided into two groups according to the variations in serum *β*-hCG levels. Ascension group was characterized by stable patients with unruptured tubal pregnancy showing no symptoms and presenting rising *β*-hCG levels in an interval of 48 h between the two measurements, and the decline group was characterized by stable patients presenting *β*-hCG levels declining in an interval of 48 h between the two measurements.

Diagnosis of EP was based on the protocol used in the Department of Obstetrics of UNIFESP and is based on an association between the patient's prior clinical history, their gynecological examination, and results of transvaginal ultrasound (TVUS), as well as on the quantitative *β*-hCG levels. All patients initially underwent the noninvasive diagnosis of EP based on TVUS and measurement of *β*-hCG. The goal was to take two measurements of serum *β*-hCG in an interval of 48 h before surgery. After obtaining the curve of *β*-hCG all patients underwent surgery. The diagnosis was confirmed intraoperatively and salpingectomy was performed. An experienced pathologist (JNS) who was blinded to the clinical and laboratory characteristics of patients carried out immunohistochemical analysis. To reduce intraobserver variability all analyses were conducted at a time only in the following ways.

### 2.1. Collection of Fraction Beta-Human Chorionic Gonadotropin

Ten (10) mL of venous blood was collected in a plain tube. Serum separated from the blood was used to confirm the diagnosis of ectopic pregnancy by detecting the increase or decrease in *β*-hCG levels using enzyme immunoassay (AIAPACOTE *β*-hCG, Tosoh Medic, Inc., Yamaguchi, Japan).

### 2.2. Immunohistochemical Analysis

Tissue samples obtained from salpingectomy after fixation in xylene and processing in graded alcohol were embedded in paraffin blocks and sectioned in serial sections of 3-micron thickness and mounted on positively charged slides for immunohistochemical analysis. Sections were deparaffinized in xylene, rehydrated in series of graded alcohol solution, and finally rinsed in water. After blocking the endogenous activity with 3% hydrogen peroxide solution for 15 minutes at room temperature, sections were washed in 1X Tris buffered saline (TBS, pH 7.4). 1X TBS was used for all the dilution of antibodies and washes during the immunohistochemical staining process. Avidin-biotin peroxidase complex (ABC) immunostaining kit was used, according to manufacturer's protocol (Vector Stain Elite, Vector Laboratories, Peterborough, UK).

### 2.3. Ki-67: A Cell Proliferation Marker

First the proliferative activity of the trophoblastin cut sections was evaluated by using the antibody to cytokeratin 7 and then by using Ki-67 antibody. Immunohistochemical staining for Ki-67 was carried out according to the specification described in the universal peroxidase kit (Immunotech, Marseille, France). Positive control used for Ki-67 was tonsil and the negative control was devoid of the primary antibody. Sections were deparaffinized in xylene, rehydrated in series of graded alcohol solution, and finally rinsed in water. Sections were treated with normal serum followed by primary antibody MIB-1 (Immunotech) and stained with hematoxylin. A cell was considered positive when part or all of the nuclei are colored. The nuclei staining analysis divided the cases into three groups: grade I (less than 1/3 of stained nuclei), grade II (from 1/3 to 2/3 of stained nuclei), and grade III (more than 2/3 of the stained nuclei).

### 2.4. VEGF: An Angiogenesis Marker

VEGF expression in the section was carried out using polyclonal anti-VEGF antibody (1 : 200 dilution, rabbit, Santa Cruz Biotechnology). Positive control used for VEGF was placenta and negative control was devoid of the primary antibody. Slides were analyzed by light microscopy to determine reaction positivity. In this analysis, patients were divided into three groups: grade I: less than 1/3 of the stained cytoplasm, grade II: 1/3 to 2/3 of the stained cytoplasm, and grade III: more than 2/3 of the stained cytoplasm.

### 2.5. Statistical Analysis

Obtained data were summarized using the mean, standard deviation, median, minimum, and maximum values for numeric variables. Data analysis was performed employing the program Minitab Inc., version 1.16, 2010. Statistical comparison between groups I, II, and III on the basis of nuclei staining and VEGF staining and categorical variables was performed using chi-square, ANOVA, and Kruskal-Wallis tests. The result was considered statistically significant when the *P* value was less than 0.05. The number of cases was based on two previous articles that assessed Ki-67 and EP [4, 5]. We did not realize sample size calculation.

## 3. Results

The mean age of patients was 27.6 years (range 24–40 years), mean gestational age at the time of hospitalization was 7.72 weeks (range 5 weeks and 3 days to 10 weeks), and the overall mean level of *β*-hCG was 6055.59 mIU/mL on the day of admission (ranging from 415 mIU/mL to 24.590 mIU/mL).

**Figure 1:**
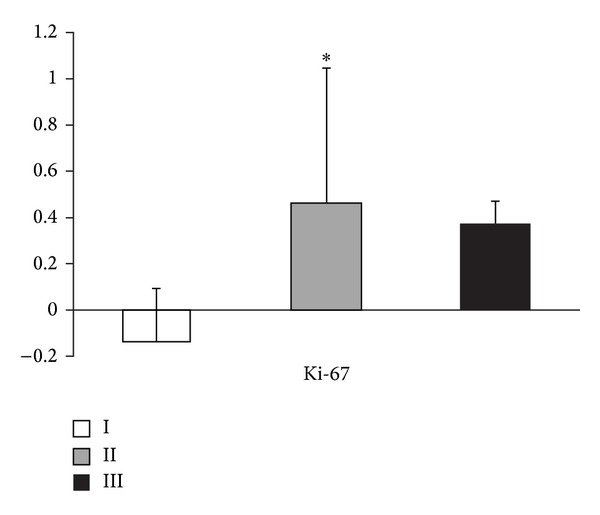
Mean variation of serum *β*-hCG in accordance with the immunoreactivity of cells labeled by Ki-67. Values are expressed as mean change in percentage (%) with their standard deviations. *P = 0.030.

The mean variation of *β*-hCG levels in an interval of 48 h prior to surgery in patients with trophoblastic cell proliferation assessed by Ki-67 showed a decline of 13.46% in grade I (8 cases), a rise of 45.99% in grade II (7 cases), and a rise of 36.68% in grade III (3 cases) in cell proliferative activity (*P* = 0.030) (Table 1 and Figure 1).

**Figure 2:**
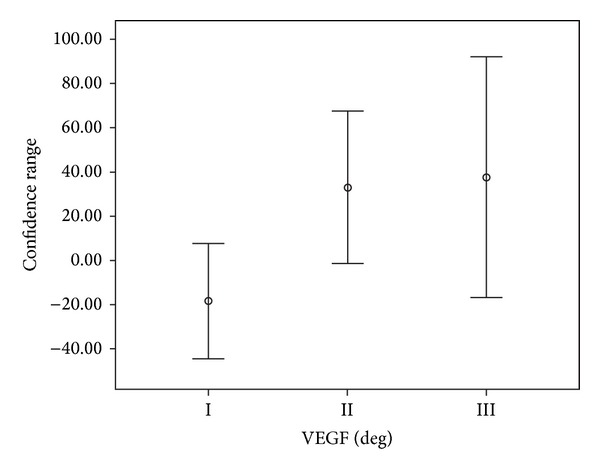
Mean variation of serum *β*-hCG in accordance with the immunoreactivity of cells labeled by VEGF.

In the assessment of angiogenesis by VEGF, of the 18 cases analyzed, the average variation of *β*-hCG in 48 hours showed a decline of 18.35% in grade I, a rise of 32.95% in grade II, and ascension of 37.55% in grade III (*P* = 0.0477) (Table 2 and Figure 2).

## 4. Discussion

With respect to cell proliferation assessed by Ki-67 [4, 5] showed that the more pronounced the increase in *β*-hCG levels, greater is the biological activity of the trophoblast. Based on this assumption and trying to understand the growth and activity of trophoblast in tubal wall, we compared the ascension and decline groups in cell proliferation assessed by Ki-67. We found that 10 cases (55.55%) had a mean increase of 41.33% of the *β*-hCG levels in 48 hours, with cell proliferation grades II and III, and 8 cases (44.45%) had a mean decrease of 13.46% of the *β*-hCG levels in the same period, with grade I cell proliferation, outlining a statistically significant correlation (*P* = 0.030) between cell proliferation and variation of serum *β*-hCG levels in 48 h.

Klein et al. [4] and Kiss et al. [5] found a variation of 77.3% and 75.3%, respectively, in increment of *β*-hCG levels in 48 h, in cases with grade III cell proliferation, demonstrating that an active trophoblast results in elevation in *β*-hCG levels within 48 h prior to surgery. Studies have shown that increase in *β*-hCG level in 48 hours is associated with failure of systemic treatment with MTX [12–14]. da Costa Soares et al. [13] demonstrated that when the variation of *β*-hCG levels within 48 hours is greater than 36.28% ± 44.31% the therapy with MTX had a higher risk of failure.

In our study, we observed that when the variation of *β*-hCG levels within 48 hours was greater than 44.45% patients showed trophoblastic cell proliferation activity of grades II and III, assessed by Ki-67, correlating our results with that of da Costa Soares et al. [13]. The American Society for Reproductive Medicine considers an increase of more than 50% of *β*-hCG level within 48 hours before treatment as an indicator for failure of medical treatment with MTX [15].

In cases where initial *β*-hCG level is low but show rapid increase in *β*-hCG levels in 48 hours, there is greater chance of tubal rupture, demonstrating an active trophoblast. This information should be considered as an option for MTX treatment to avoid persistence of trophoblastic tissue and treatment failure [15].

The main inclusion criterion for expectant management of EP is declining *β*-hCG levels in 48 hours [12]. Mavrelos et al. [16] have shown expectant management in 144 tubal pregnancies with *β*-hCG level less than 1500 mIU/mL. They observed that in 80 cases the *β*-hCG level declined in the second test, whereas in 64 patients the *β*-hCG level increased. The success obtained in patients with a decline in *β*-hCG was 88.8% and the rise of *β*-hCG level was 51.6%. Therefore, the decline of *β*-hCG level in 48 hours is predictive of success for expectant management. Data obtained from our study shows that most of the patients with declining *β*-hCG levels in 48 hours also showed low trophoblastic cell proliferation assessed by Ki-67, equivalent to grade I (mean decline in *β*-hCG levels in 48 hours was 13.46%), which gives better expectant management of EP and does not need surgical treatment.

EP has a curve evolution with the rise and decline of *β*-hCG level, where the peak of the curve, in other words, the time when the *β*-hCG levels reach their maximum values, is decisive in the evolution of case because at this point two things can happen: the case can progress to tubal rupture with acute hemorrhage in abdomen or can start the process of involution and reabsorption, evolving to spontaneous healing.

The trophoblast implantation into the tube occurs in a hypoxic environment; this event triggers the mechanism that stimulates the production of VEGF [6]. Previous studies have observed a high expression of VEGF in syncytiotrophoblast and cytotrophoblast, suggesting the active role of VEGF in the infiltration of trophoblast during implantation [7, 8].

In order to understand the correlation between VEGF and *β*-hCG level, we performed immunohistochemical analysis comparing this factor with the groups of patients with the rise and decline of *β*-hCG level in 48 hours. In the assessment of angiogenesis by VEGF, we observed that patients with the mean rise of 37.55% of *β*-hCG levels had increased angiogenesis and patients with mean decline of 18.35% of *β*-hCG levels showed low angiogenesis, with a statistical significance (*P* = 0.0477).

Cabar et al. [8] performed a study evaluating VEGF in maternal serum and found that VEGF levels around 297.2 pg/mL predicted an infiltration of the trophoblast until the mucosa, with 100% sensitivity and positive predictive value, and levels above 440.1 pg/mL predicted a complete infiltration of the tubal wall and postulated that a high cellular production of VEGF allows deeper infiltration of trophoblast. Correlating our findings with previous studies [6–8], we can infer that VEGF is an angiogenic factor responsible for the deployment and therefore has great importance as a valuable tool in early diagnosis of EP and in its therapeutic orientation.

Despite small sample size, the results of this study reinforce the importance of the correlation between *β*-hCG level and the trophoblastic activity. The variation of *β*-hCG level in 48 hours was directly related to the trophoblastic cell proliferation assessed by Ki-67, and mean *β*-hCG level in 48 hours was related to angiogenesis assessed by VEGF.

In this research we seek to unravel the immunohistochemical factors associated with failure of conservative treatment of ectopic pregnancy. Relevant information from this study is that the curve of evolution of *β*-hCG before instituting treatment reflects the degree of cell proliferation, essential condition for clinical indication. The microscopic demonstration of the phenomena observed with clinical experience of conservative treatment is of great value to better selection of cases for this type of treatment in tubal pregnancy avoiding failures and sparing patients the therapy failure.

## Figures and Tables

**Table 1:** Mean variation of serum *β*-hCG in 48 hours prior to treatment of ectopic pregnancy according to the degree of cell proliferation measured by Ki-67. Values are expressed as mean percentage change (%) with standard deviations (SD), minimum, and maximum values.

Ki-67	*N* (*n* = 18)	Mean variation of *β*-hCG at 48 h (%) ± SD (min–max)
I	8	−13.46 ± 22.59 (−56.86–8.42)
II	7	+45.99 ± 58.34 (−12.68–118.88)
III	3	+36.68 ± 10.27 (27.35–47.70)

*P* = 0.03.

**Table 2:** Change of serum *β*-hCG in 48 hours according to the degree of angiogenesis by VEGF evaluated. Values expressed as mean percentage of variation (%) with standard deviations (SD), minimum, and maximum values.

VEGF	*N* (*n* = 18)	Mean variation of *β*-hCG at 48 h (%) ± SD (min–max)
I	6	−18.35 ± 24.49 (−56.87–8.42)
II	5	+32.95 ± 27.70 (2.26–77.50)
III	7	+37.55 ± 58.79 (−12.68–118.80)

*P = 0.047. *P value obtained by the Kruskal-Wallis test.
